# Fish Bone Derived Bi-Phasic Calcium Phosphate Coatings Fabricated by Pulsed Laser Deposition for Biomedical Applications

**DOI:** 10.3390/md18120623

**Published:** 2020-12-07

**Authors:** Gianina Popescu-Pelin, Carmen Ristoscu, Liviu Duta, Iuliana Pasuk, George E. Stan, Miruna Silvia Stan, Marcela Popa, Mariana C. Chifiriuc, Claudiu Hapenciuc, Faik N. Oktar, Anca Nicarel, Ion N. Mihailescu

**Affiliations:** 1 National Institute for Lasers, Plasma and Radiation Physics, RO-077125 Magurele, Romania; gianina.popescu@inflpr.ro (G.P.-P.); carmen.ristoscu@inflpr.ro (C.R.); liviu.duta@inflpr.ro (L.D.); hapenciuc.claudiu@inflpr.ro (C.H.); 2 National Institute of Materials Physics, RO-077125 Magurele, Romania; iuliana.pasuk@infim.ro (I.P.); george_stan@infim.ro (G.E.S.); 3 Department of Biochemistry and Molecular Biology, Faculty of Biology, University of Bucharest, RO-050095 Bucharest, Romania; miruna.stan@bio.unibuc.ro; 4 Microbiology Department, Faculty of Biology, University of Bucharest, RO-060101 Bucharest, Romania; marcela.popa@bio.unibuc.ro (M.P.); carmen.chifiriuc@bio.unibuc.ro (M.C.C.); 5 Research Institute of the University of Bucharest (ICUB), University of Bucharest, RO-050095 Bucharest, Romania; 6 Academy of Romanian Scientists, Ilfov Street no. 3, RO-050711 Bucharest, Romania; 7 Department of Bioengineering, Faculty of Engineering, Goztepe Campus, University of Marmara, Kadikoy, 34722 Istanbul, Turkey; foktar@marmara.edu.tr; 8 Center for Nanotechnology & Biomaterials Research, Goztepe Campus, University of Marmara, Kadikoy, 34722 Istanbul, Turkey; 9 Physics Department, University of Bucharest, RO-077125 Magurele, Romania; anca_nicarel@yahoo.com

**Keywords:** antimicrobial coatings, advanced implants, nosocomial infections prevention, cytocompatibility, sustainable resources, PLD

## Abstract

We report on new biomaterials with promising bone and cartilage regeneration potential, from sustainable, cheap resources of fish origin. Thin films were fabricated from fish bone-derived bi-phasic calcium phosphate targets *via* pulsed laser deposition with a KrF * excimer laser source (λ = 248 nm, τ_FWHM_ ≤ 25 ns). Targets and deposited nanostructures were characterized by SEM and XRD, as well as by Energy Dispersive X-ray (EDX) and FTIR spectroscopy. Films were next assessed in vitro by dedicated cytocompatibility and antimicrobial assays. Films were Ca-deficient and contained a significant fraction of β-tricalcium phosphate apart from hydroxyapatite, which could contribute to an increased solubility and an improved biocompatibility for bone regeneration applications. The deposited structures were biocompatible as confirmed by the lack of cytotoxicity on human gingival fibroblast cells, making them promising for fast osseointegration implants. Pulsed laser deposition (PLD) coatings inhibited the microbial adhesion and/or the subsequent biofilm development. A persistent protection against bacterial colonization (*Escherichia coli*) was demonstrated for at least 72 h, probably due to the release of the native trace elements (i.e., Na, Mg, Si, and/or S) from fish bones. Progress is therefore expected in the realm of multifunctional thin film biomaterials, combining antimicrobial, anti-inflammatory, and regenerative properties for advanced implant coatings and nosocomial infections prevention applications.

## 1. Introduction

Calcium phosphates (CaPs) stand for the bioceramic materials most frequently used in medicine, in particular in orthopaedics and dentistry, for the treatment of various bone fractures and defects, craniomaxillofacial reconstruction, or fabrication of coatings for numerous metallic implants and porous scaffolds [1,2]. Special focus was put on hydroxyapatite (HA) and β-tricalcium phosphate (β-TCP), which are the most frequently used CaPs in biomedical applications [3,4]. The synthesis of CaPs can proceed by chemical routes, starting either from inorganic reagents, or natural resources [5,6,7].

Synthetic HA, Ca_10_(PO_4_)_6_(OH)_2_, along with bioglass [8,9], are among the most studied bioactive materials in medicine [4,10]. The fabrication of synthetic HA is generally achieved by sol-gel [11], co-precipitation [12], and hydrothermal [13] routes. Nevertheless, these approaches imply the use of complex processes that could generate pollutant chemical wastes, harmful for the environment. Moreover, they are time-consuming methods and involve rather high production costs. Moreover, the chemistry of synthetic HA does not entirely reproduce the composition of bone mineral phase [14]. One should warn here that the available mineral resources are nowadays threatened and strained by the rapid demographic increase and economic growth. The access to unconventional, sustainable resources is therefore needed and encouraged for future economic development and prosperity [7]. One simple, reliable, and highly productive alternative to classical chemical routes [15] is to fabricate HA materials from renewable resources often treated as food industry by-products only [6]. Biogenic animal and fish bones are an excellent opportunity in this respect.

Biphasic calcium phosphates (BCPs) represent a group of bone substitute materials most commonly consisting of a mixture of two CaP phases, in different concentrations, i.e., the soluble (β-TCP) bringing the advantage of a favorable degradation rate and the stable (HA) one, ensuring stability and good mechanical properties [16]. As expected, BCPs are exhibiting important advantages over single-phase formulations (e.g., pure β-TCP, HA, or other CaP bioceramics). The control of the biocompatibility and biodegradability warrants, in this case, a good stability while promoting an effective bone ingrowth [16,17,18]. One can therefore fabricate, by proper matching the phase concentrations, BCP materials for large-scale bone defects restoration in high-load bearing areas, or as customized parts, which preserve their configuration over prolonged exploitation times [19].

Biological CaPs, of fish origin or obtained from cortical or cancellous animal bones, contain trace elements (such as Na, Si, Mg, K, F, or C), also present in human bones [5,7]. Besides carbonate ions, these elements exist in trace concentrations ≤ 1 wt.% [20]. The trace elements play a key-role in the proper adjustment of biological properties of CaP materials such as the solubility or surface chemistry and morphology, to keep compatible with the natural human bone [21].

In particular, Na is ubiquitously found in the bone mineral component, with an important role in resorption and cell adhesion processes [22,23]. Si proved also to be an essential trace element for bone and cartilage growth and development, extending the biological activity as compared to stoichiometric counterparts [24]. Mg is involved in the qualitative changes of bone matrix, controlling the bone fragility [24]. The simultaneous incorporation of Si and Mg was proposed for the synthesis of CaP materials with biological apatite-like composition [24,25].

Besides the huge cut of production costs, the fabrication of bioapatite from natural resources (i.e., bones) secures the preservation of especially composition and structure of the source material [26], and allows for a superior biomimicry. The production of quite cheap medical devices becomes accessible on these bases [27,28,29,30].

HA is presently fabricated from renewable resources by either freeze-dried methods or diluted acid treatments [31]. These approaches cannot unfortunately guarantee the HA production free of residual deadly prions, which can survive during the process. To surpass this drawback and eliminate the risk of diseases transmission, calcination at 850 °C (for 4‒6 h) was applied. The complete elimination of the bone organic part together with the conservation of the inorganic one, including trace elements, is this way expected. No prions can survive such sintering temperatures and treatment times [5]. The method respects the EU settlement no. 722/2012 and ISO no. 22442/2007, which regulate the management of health security issues when working with products derived from tissues of animal origin.

The most important primary natural reservoirs of biological HA are either (i) biogenic products or organisms from the aquatic environment—egg-shells [32], corals [33], various types of sea-shells [34] or fish (tuna and sword fish [15], turbot [35], salmon [36], mackerel, gilded catfish, and spotted sorubim [37]) bones, or (ii) terrestrial organisms, such as mammalian (bovine [7,38,39], sheep [5], goat [40,41], pig [42]), or bird [40] bones. Among above-mentioned HA sustainable resources, fish bones stand for the most abundant (with an annual consumption of fish and shellfish in excess of 91 million tons [15]) and low-cost alternative for CaP bioceramic production [6]. Moreover, fish bones are richer in oligo-elements (in both number and quantity), in comparison to terrestrial animals’ bones, due to the gradual concentration decrease of many elements in the Earth’s crust down to 20 m and deeper, while they are still present in water, especially seas and oceans.

One should also emphasize upon the low risk of disease transmission due to (i) the evolutionary distance between fish and humans, and (ii) different habitats [43]. The valorization of fishery by-products, especially bones, is expected to essentially contribute to a sustainable and efficient use of fish resources [44], and to a substantial reduction of pollution and waste management costs. Considerable scientific and technological efforts were therefore recently focused on the large potential of these by-products as sources of bioactive compounds with wide pharmaceutical and biotechnological applications [44].

We report herewith on the fabrication of biological BCP-type coatings, by pulsed laser deposition (PLD), from bioceramic targets prepared from *Sparus aurata* (sea bream) and *Salmo salar* (salmon) fish bones. Physical–chemical and biological features of the coatings were studied by complementary methods, and the results are discussed from the perspective of the production of a new generation of advanced implants and protective layers against medical devices associated infections.

## 2. Results and Discussion

For an easier track and understanding of reported results, the codes of prepared and studied samples were collected in Table 1.

### 2.1. Morphological and Compositional Investigations

**Figure 1:**
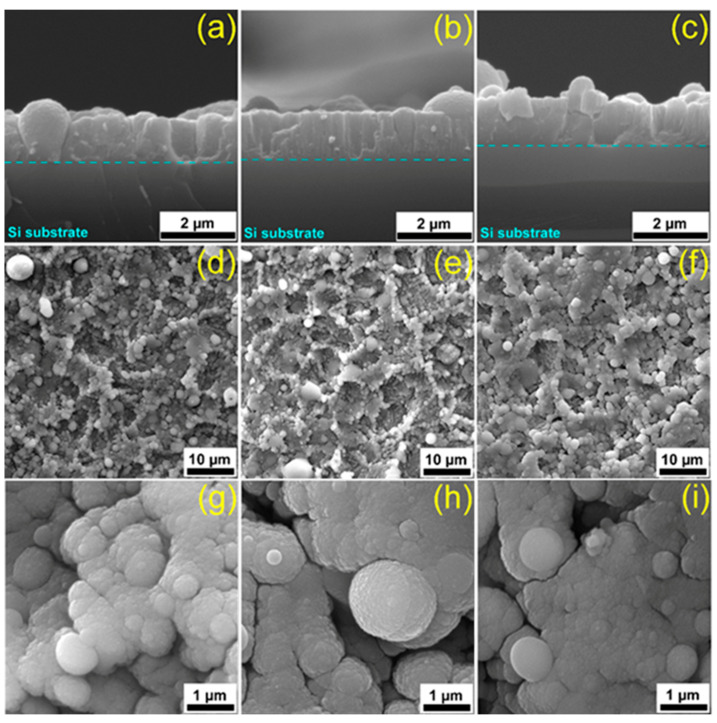
Cross-section (**a**–**c**) and top-view (**d**–**h**) SEM micrographs of (**a**,**d**,**g**) HA_syn_, (**b**,**e**,**i**) Spa-BCP, and (**c**,**f**,**h**) Sal-BCP pulsed laser deposition (PLD) coatings, respectively.

Figure 1 displays typical cross-sectional and top-view SEM images for HA_syn_, Spa-BCP, and Sal-BCP films after the post-deposition thermal treatment.

Cross-sectional SEM images (Figure 1a–c) of coatings provide key-information on both the growth morphology and interface to the substrate. An intimate contact between the film and the substrate was observed for all types of PLD samples. The average thickness of coatings was estimated based on multiple surveys along the cross-section and the inferred values were (1.3 ± 0.3) µm for HA_syn_, (1.1 ± 0.1) µm for Spa-BCP, and (1.05 ± 0.1) µm for Sal-BCP films. The corresponding deposition rates were thereby of (0.085 ± 0.017) nm/pulse for HA_syn_, (0.073 ± 0.002) nm/pulse for Spa-BCP, and (0.07 ± 0.007) nm/pulse for Sal-BCP.

The top-view micrographs collected at low SEM magnification (Figure 1d–f) showed a coarse morphology, with characteristic quasi-spheroidal particulates (droplets), typical to HA coatings synthesized by PLD [5,45,46]. The morphological comparison between different PLD coating specimens evidenced similar topologies in form of cauliflower-like aggregates. At higher SEM magnification (Figure 1g–i), it was observed that these large clusters are in fact constituted by grains with relatively well-defined boundaries, with diameters within the (0.09–2.5) µm range.

**Figure 2:**
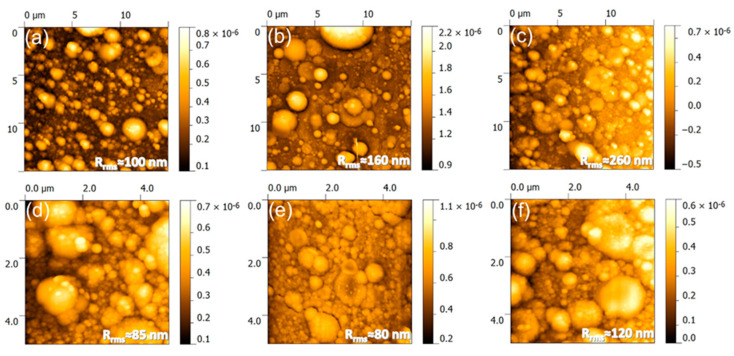
Atomic force microscopy (AFM) images recorded on (**a**–**c**) 15 × 15 µm^2^ and (**d**–**f**) 5 × 5 µm^2^ areas of the (**a**,**d**) HA_syn_, (**b**,**e**) Spa-BCP, and (**c**,**f**) Sal-BCP PLD coatings, respectively. The root mean square roughness (R_rms_) values are given on each micrograph.

The atomic force microscopy (AFM) analyses (Figure 2) endorse these observations. Unevenly spread-out nanoparticles of (50–200) nm were observed on the areas in-between the micro-aggregates. The combination of micro- and nano-scale particulates results in a rough PLD films surface, with root mean squared roughness (R_rms_) values in excess of 80 nm. The film roughness, irrespective of AFM scanned sample areas, increased in the order HA_syn_ < Spa-BCP < Sal-BCP, thereby being seemingly influenced by the content of β-TCP.

Noteworthy, rough surfaces with micron-scale features are known to have a favorable effect on osseointegration. Namely, such surfaces can stimulate both the proteins adhesion (entailed in the osteoblast proliferation process) and the adhesion and differentiation of bone cells [47].

The elemental composition and Ca/P molar ratio (mean ± SD) of PLD targets and coatings are displayed comparatively in Table 2, based on the recorded EDX spectroscopy data. For fish bone (targets and films) samples, traces of Na, Mg, Si, and S (for Spa-BCP) and Na, Mg, and Si (for Sal-BCP) were detected, besides Ca, P, and O (the main constituents of the mineral vertebrata bone) (Table 2). As known, Na, Mg, and Si are characteristic natural dopants of bone mineral [48,49].

The HA_syn_ target has a Ca/P molar ratio of ~1.66, very close to the stoichiometric value of 1.67. At variance, the other two biogenic materials elicited lower Ca/P values (i.e., Spa-BCP ≈ 1.65 and Sal-BCP ≈ 1.55). This suggested the partial decomposition of HA into β-TCP (with a theoretical Ca/P molar ratio of 1.5) during the powder fabrication process or/and a calcium deficient HA, characteristic to the bone mineral phase [50]. The Ca/P molar ratio of all PLD films had lower values with respect to corresponding parent materials. The calcium-deficient PLD coatings synthesized from mammalian bone-derived CaP ceramics has been previously reported [5].

It is difficult to advance at this point a definite, unique explanation for the observed trend. Ca was partially eliminated during, and most probably, after film deposition, resulting in a Ca-deficient structure [51]. Nevertheless, to the difference of stoichiometric HA, the biological HAs contain substituent cation trace elements, and are therefore Ca-deficient [50]. One should point here that Ca-deficient HA is more soluble than the stoichiometric one, which makes it easier resorbable. Indeed, the high stability of stoichiometric HA is considered a drawback, which is however partially surpassed by the presence of Ca-deficient material [52].

Dedicated experiments demonstrated that Ca-deficient HA could be used for in vivo studies due to the high specific area. It was therefore suggested that Ca-deficient HA could be a superior replacement solution in orthopedic surgery [50].

### 2.2. Structural Characterization

**Figure 3:**
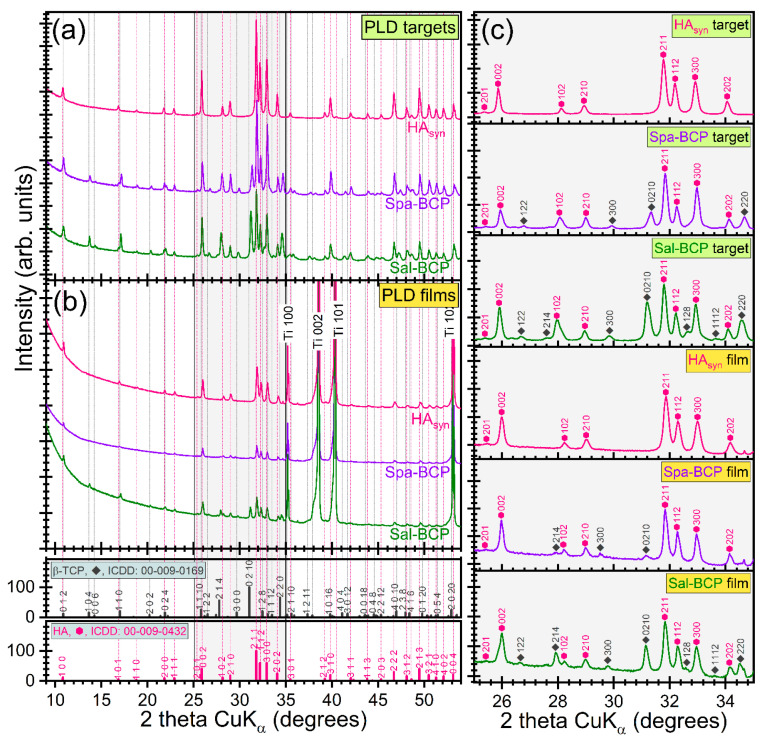
Comparison of XRD patterns of PLD (**a**) targets and (**b**) thin films. (**c**) XRD diagrams (corresponding to each type of target and film) zoomed in angular region 2θ = 25–35° to emphasize the evolution of the prominent hydroxyapatite (HA) and β-tricalcium phosphate (β-TCP) diffraction maxima.

The XRD patterns of PLD targets and films are displayed comparatively in Figure 3a,b. As an important note, the PLD process well preserved the structural phases and overall crystalline status. To the difference of HA_syn_ (target and film), where solely pure, well crystallized and randomly oriented hexagonal HA phase was detected (ICDD-PDF4: 00-009-0432), a series of supplemental diffraction peaks associated to the β-TCP phase (ICDD-PDF4: 00-009-0169) were present in the case of Spa-BCP and Sal-BCP samples (targets and films). For a better visualization of the evolution of the two CaP phases for each sample, zoomed relevant regions of the patterns (2θ = 25–35°) are displayed in Figure 3c. Due to micron-sized thickness of PLD films, the reflections of the Ti substrate (ICDD-PDF4: 00-044-1294) are also visible.

**Figure 4:**
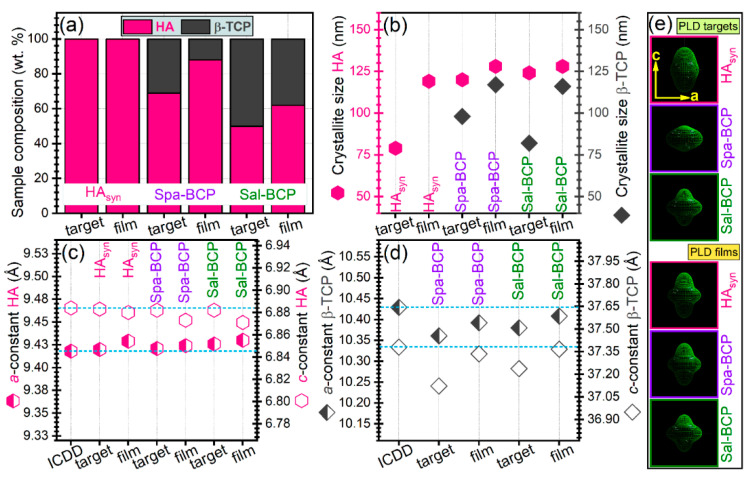
(**a**) Phase composition, (**b**) mean crystallite size of HA and β-TCP phases, (**c**,**d**) evolution of the *a*- and *c*-axis lattice parameters for (**c**) HA and (**d**) β-TCP phases. (**e**) HA “composite crystallite” shapes as determined by MAUD software, according to the Popa approach [53]. Note: the dashed horizontal lines in (**c**,**d**) mark the ICDD values of parameters. The error bars of refined structural parameters (provided by the fitting software) are not figured, since they were too small to be well visualized.

Quantitative structural assessments were next inferred by Rietveld pattern fitting, and graphically presented in Figure 4. A higher β-TCP content (~50 wt.%) was revealed in the case of the Sal-BCP target with respect to the Spa-derived one (~31 wt.%). A concentration decrease of the β-TCP content was noticed for both Sal-BCP and Spa-BCP films, down to ~38 and ~12 wt.%, respectively (Figure 4a).

The mean crystallite size of HA phase got values of (~120–128) nm for both targets and films (Figure 4b), irrespective of the pure or bi-phasic nature of the sample. The average crystallite size of the β-TCP component appears however larger in the films (~100–116 nm), than in the targets (~80 nm) (Figure 4b).

The evolution of the *a*- and *c*-axis lattice parameters for HA and β-TCP phases is given in Figure 4c,d. In the case of HA component (in both targets and films), minor lattice parameter modifications only, were observed with respect to the ICDD-PDF4: 00-009-0432 reference file (Figure 4c). A similar trend was highlighted for the β-TCP phase in PLD films (Figure 4d). A more consistent modification of lattice parameters (but, never exceeding more than 1% with respect to the values listed in the corresponding ICDD-PDF4: 00-009-0169 file) was noticed for the β-TCP component of the PLD targets (Figure 4d). The modifications of lattice parameters of the CaP phases from target to film reflect small changes of the average stoichiometry, as shown by the changes of the overall Ca/P ratio (Table 2).

The “composite crystallite” shapes inferred by MAUD software are presented in Figure 4e. They point to the crystallographic orientation dependence of HA crystallites size in terms of the aspect ratio in the *c* to the *a* crystallographic directions. Thus, the “composite crystallite” of the isotropic crystallite is spherical [53]. The results in Figure 4e showed that the crystallites are on average: (i) elongated along the c direction for the HA_syn_ target; (ii) flattened in the c direction for the Spa-BCP target; and (iii) mixtures of elongated and flattened crystallites for the Sal-BCP one.

**Figure 5:**
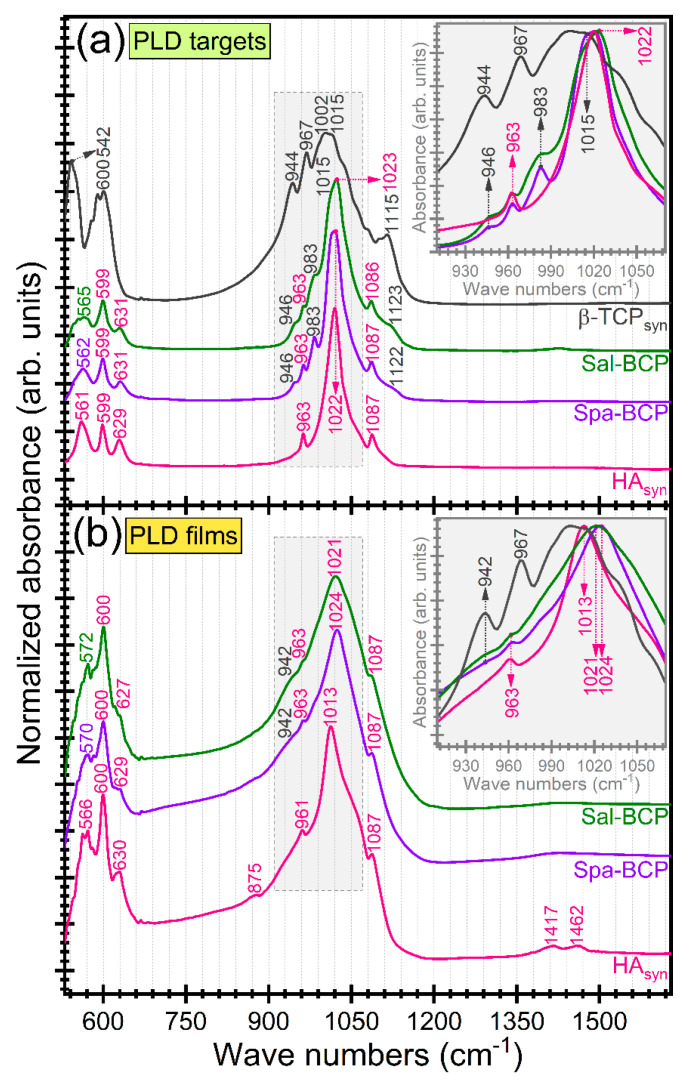
FTIR-ATR spectra of PLD (**a**) targets and (**b**) derived coatings. Insets: zoomed regions of symmetric and asymmetric stretching vibrations of phosphate groups in HA and β-TCP. For comparison, the FTIR-ATR spectrum of pure (synthetic) β-TCP powder (Sigma-Aldrich) is presented in dark-grey color.

FTIR-ATR spectra of PLD targets and films are displayed in Figure 5. In addition, the reference IR spectrum of pure β-TCP (Sigma-Aldrich) is inserted in Figure 5a. The complete vibration bands assignment was collected in Table 3. The slight shift of the IR bands position between targets and films could be ascribed to the structural alterations occurring during the PLD transfer and reassembling in form of thin coating.

The absorption IR bands at lower wave numbers (~600–599, 572–561, and 542 cm^−1^, respectively) are attributed to the ν_4_ asymmetric bending mode of the orthophosphate units in both HA and β-TCP-type phases [5,57,58,59]. The presence of β-TCP phase in the fish-bone target materials was supported by the incidence of the three distinct stretching bands peaking at ~1153–1123, 983–967, and 946–944 cm^−1^, respectively [54,55,56]. The broader aspect of the IR bands, in the case of PLD films, impeded a facile discrimination of the three trademark IR bands of β-TCP. They were indeed obscured by the characteristic symmetric (~963–961 cm^−1^) and triple degenerated asymmetric (~1087 and 1024–1013 cm^−1^) bands of orthophosphate functional groups in a HA-type structure [5,57,58,59].

The presence of structural hydroxyl groups, distinctive to HA phase was confirmed by the advent of the librational band at 631–627 cm^−1^ [57,58,59]. In the case of PLD films, this band was well-preserved for the HA_syn_, but strongly decreased in intensity for the Spa- and Sal-based BCP structures, pointing to an acute de-hydroxylation of the HA counterpart.

The presence of the ν_2_ out-of-plane bending (~877 cm^−1^) and ν_3_ asymmetric stretching (~1462 and 1417 cm^−1^) of HA_syn_ films is indicative for the B-type carbonatation (with the carbonate groups substituting the orthophosphate groups into the HA crystalline lattice) [57,58,59]. The presence of carbonate substituting groups was signaled out for all target materials, but in a significantly lower content (data not shown here).

A more pronounced carbonatation (with respect to the target material) was evidenced in the case of the fish-bone derived films. This carbonatation increase, observed for films, was expected for structures crystallized by heat-treatments carried out under ambient air enriched with water vapors. The improved behavior of carbonated HA materials with respect to pure HA was already reported [60,61,62]. It is due to the higher in situ solubility, able to enhance the osseointegration processes. The increased resorption speed of carbonated HAs could be ascribed to the weaker Ca–CO_3_ bonds compared to the Ca–PO_4_ ones [63]. It is therefore more relevant to test the biological performance of the fish-bone derived BCP materials (containing a highly soluble β-TCP component) against a potent control sample (such as the carbonated HA_syn_ films).

### 2.3. Film Bonding Strength Evaluation

**Figure 6:**
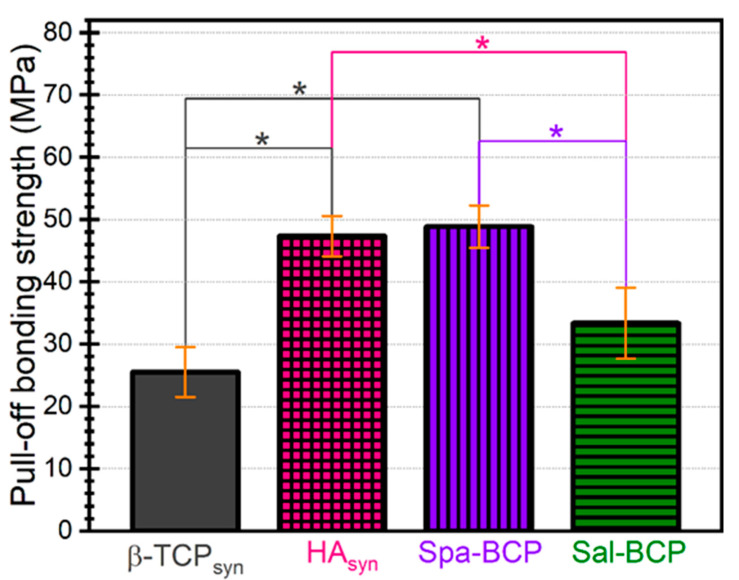
Pull-off test results in the case of the calcium phosphates (CaP)-based coatings deposited by PLD onto Ti disks. Note: * *p* < 0.05, one-way ANOVA followed by Tukey-Kramer post-hoc test.

The pull-off tests (Figure 6) indicated dissimilar performance of the PLD CaP-based samples. β-TCP and Sal-BCP coatings (with a higher concentration of β-TCP) were cleanly detached from the Ti substrate, yielding bonding strength values of ~25 and ~33 MPa, respectively. For HA_syn_ and Spa-BCP (with a lower concentration of β-TCP) coatings, the pull-off detachment values were situated in a narrow range of (47–49) MPa, with the fracturing occurring each time in the volume of the adhesive used to glue the testing elements, leaving the films undamaged. It follows that the β-TCP increase in the composition of the PLD coatings can reduce the adherence to the Ti substrate. The bonding strength values were however superior to the ISO-137792 Part 2 “Coatings of Hydroxyapatite” standard of 15 MPa in the case of all tested coatings.

Nevertheless, these mechanical data should be considered with care because of the porous nature of the PLD coatings consisting of spherical particles, which can facilitate the infiltration of the adhesive substance into the film. The validity of such film adherence tests could therefore be compromised in the case of the radical penetration of glue down to the Ti substrate, which is, however, highly improbable, due to the viscous nature of the used glue.

### 2.4. In Vitro Biological Assay

#### 2.4.1. Solubility of Coatings under Biomimetic Conditions

After one day of soaking in the Dulbecco’s Modified Eagle medium (DMEM) supplemented with 10% fetal bovine serum, a larger mass loss was recorded in the case of Sal-BCP coatings (7.5 ± 2.2%) versus Spa-BCP (4 ± 0.4%). This is likely owned to the higher content of β-TCP in the Sal-BCP films (Figure 4a). Interestingly, after three days of immersion, a lower mass loss (3 ± 0.3%) and even a mass gain (6.4 ± 1.5%), were evidenced for the Sal-BCP and Spa-BCP coatings, respectively (taking as reference the initial mass of the films). This is suggesting that the precipitation of CaP might take place under homeostatic conditions, which is more pronounced for the sample with a higher content of bioactive HA phase (i.e., Spa-BCP), known to elicit improved biomineralization capacity [64]. This trend is strengthened after seven days of immersion, with both samples experiencing mass gain of ~3% and ~9% in the case of Sal-BCP- and Spa-BCP coatings, respectively.

**Figure 7:**
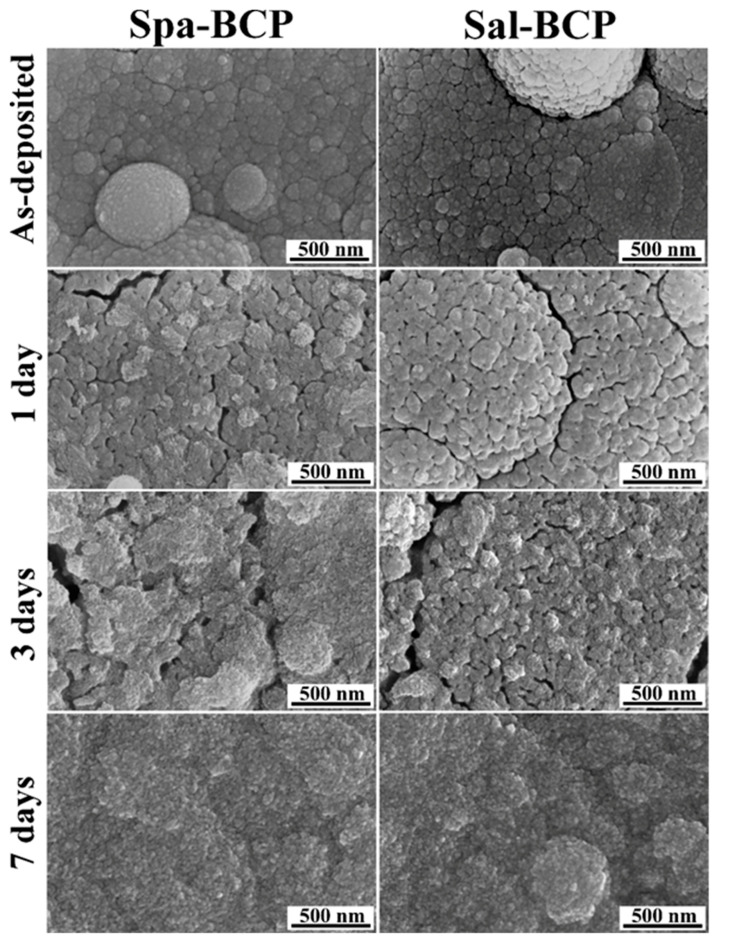
Typical SEM images of the Spa- and Sal-BCP coatings, before and after 1, 3, and 7 days of immersion in the DMEM supplemented with 10% fetal bovine serum solution under biomimetic in vitro conditions. Magnification: 100,000×.

The biomineralization (i.e., the biomimetic CaPs layer formation on the surface of the BCP coatings) hypothesis is supported by the high magnification SEM images in Figure 7. Surface morphology changes were more obvious after three days of immersion in the biomimetic medium, with the spherical PLD droplets of the initial coating covered by a thin layer of fine acicular crystals (typical to biomimetic CaPs forming in simulated body media [65,66]). The process is augmented after seven days of soaking, with the BCP coatings covered with a seemingly thicker biomimetic CaP layer of similar morphology, which completely changes the topography of the specimens.

#### 2.4.2. Cytocompatibility of Coatings

Besides optimal surgical handiness and mechanical properties, implantable biomaterials for bone and cartilage regeneration should be highly biocompatible and favorable to cellular adhesion, proliferation, and differentiation, to sustain rapid tissue regeneration after implantation.

**Figure 8:**
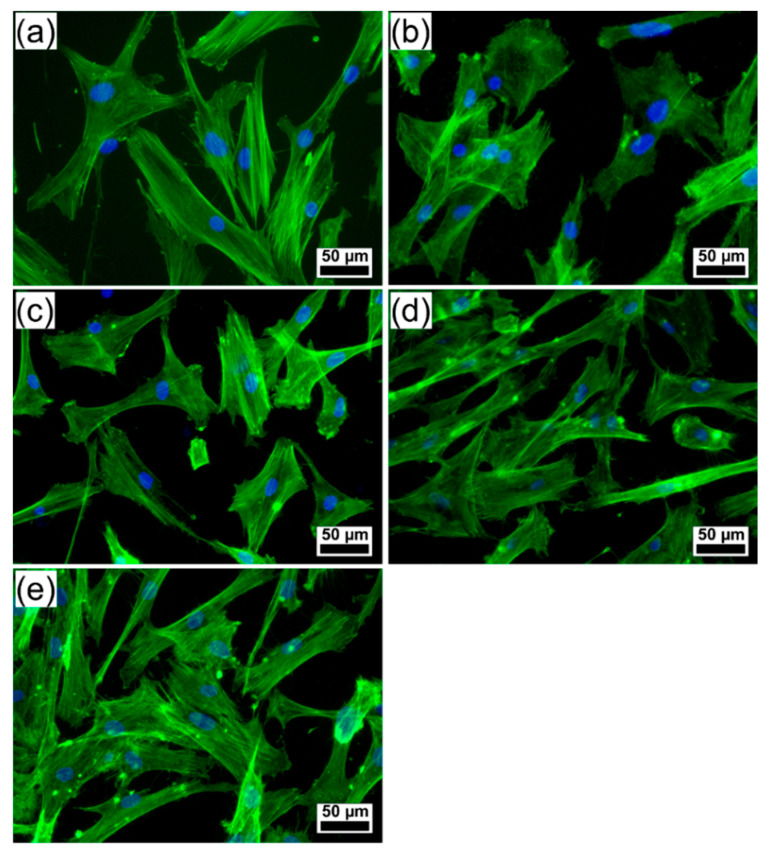
Fluorescence microscopy images showing the morphology of human gingival fibroblast cells growing on the surface of (**a**) control (polystyrene surface of the culture dish), (**b**) bare Ti, and PLD coatings: (**c**) HA_syn_, (**d**) Spa-BCP, and (**e**) Sal-BCP. (The filamentous actin was stained with phalloidin-FITC (green) and the nuclei counterstained with DAPI (blue)). Magnification bar: 50 µm.

The well-organized F-actin cytoskeleton of human gingival fibroblasts (HGF)-1 cells cultured on the surface of tested coatings (Figure 8) was examined, pointing out a good biocompatibility. The human gingival fibroblasts have been used in the study as they are in close proximity to coated dental implants, but also because they exhibit osteoblast-like features upon appropriate stimulation [67].

The fluorescence images revealed morphological features of cells attached to PLD coatings similar to control (polystyrene surface of the culture dish), with parallel actin stress fibers, designating a good cell adhesion onto the surface of tested samples. Cells grown on the surface of Spa-BCP and Sal-BCP specimens showed numerous filopodia, lamellipodia, and actin filament bundles in periphery, which facilitate the contacts between the neighboring cells and their spreading. These results suggest that the PLD coatings are capable of modulating the actin dynamics and membrane reorganization (Figure 8). Thus, the PLD modified surfaces can regulate the cytoskeleton remodeling and turnover, acting as extracellular stimuli for the gingival fibroblasts and providing the required rigidity to direct cell motility on an implant to get the best osseointegration.

**Figure 9:**
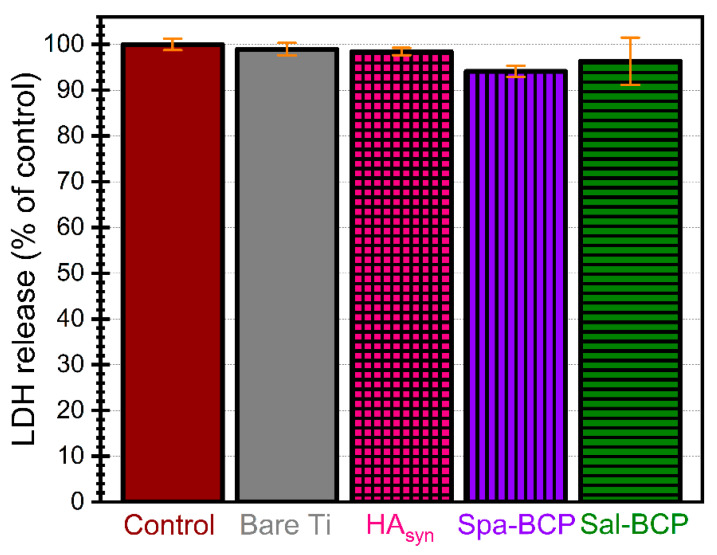
Cytotoxicity of control, bare Ti and PLD coatings as evidenced by the lactate dehydrogenase (LDH) assay after 24 h of human gingival fibroblast cells growth. Note: *p* > 0.05 for all tested situations, thereby, no statistically significant differences in terms of cytocompatibility were recorded, as indicated by performing a one-way ANOVA analysis followed by a Tukey-Kramer post-hoc test.

The evaluation of cell membrane integrity after the interaction of fibroblasts with PLD coatings was assessed by measuring the cytoplasmic lactate dehydrogenase (LDH) released into the media (Figure 9). No statistically significant changes in LDH release after 24 h exposure were observed (*p* > 0.05), confirming that none of the PLD samples damaged the integrity of the cell membranes, as no cytotoxicity was induced.

These results prove that the deposited BCP-like coatings have suitable composition and structural features, which, in turn, can ensure advantageous conditions for good cell proliferation, essential for a fast bone tissue regeneration process.

#### 2.4.3. Antibacterial Activity of Coatings

Biomaterials designed for bone and cartilage reconstruction should not only be favorable to tissue regeneration at the site of surgery, but also exhibit resistance to microbial colonization, for preventing implant associated infections. These difficult to treat infections are caused by microbial biofilms growing on medical devices, which may be responsible of exacerbated or prolonged inflammation and eventually lead to implant failure [68]. The real challenge is, therefore, to fabricate implantable multifunctional biomaterials, combining antimicrobial, anti-inflammatory, and regenerative properties. After implantation, the bacterial cells will compete with host ones to adhere to the biomaterials surface. The ideal biomaterial should thus exhibit anti-infective properties, *via* physical-chemical features, which are inhibiting the initial bacterial attachment to the biomaterial, or act by releasing antimicrobial agents that kill bacterial cells in the surrounding areas, before approaching the surface [69]. One should also emphasize that the same requirements are essential for the coatings used for prevention of nosocomial contamination of large areas in view of clinical utilization [7].

**Figure 10:**
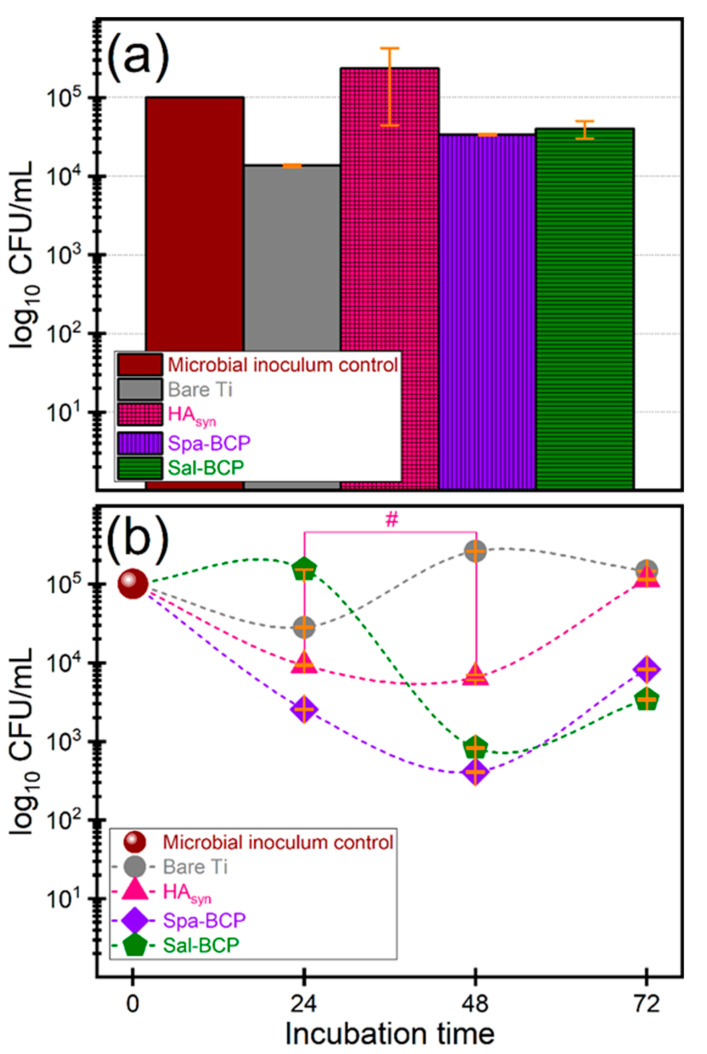
Logarithmic representation of colony forming units (CFU)/mL of the *E. coli* bacterial strains, depicting the (**a**) initial stages of microbial adhesion (10–15 min) and (**b**) microbial growth and biofilm development after different time intervals (of 24, 48 and 72 h, respectively), in the presence of the bare Ti substrates and PLD coatings of HA_syn_, Spa-BCP, and Sal-BCP. Note: # *p* > 0.05, thereby, no statistically significant difference between HA_syn_ 24 h vs. HA_syn_ 48 h, as resulted based on a one-way ANOVA analysis followed by a Tukey-Kramer post-hoc test. For all other situations *p* < 0.05.

It was thus demonstrated that the Gram-negative bacilli are causing 6–23% of all orthopedic implant infections [70]. Therefore, the antibacterial activity of the synthetized biomaterials was assessed against *Escherichia coli*, which is the most frequent Gram-negative species involved in the etiology of device-associated infections of endogenous origin. The antibacterial activity was monitored after different incubation times, to evaluate the capability of the obtained biomaterials to inhibit the initial stages of microbial adhesion, quantified after 10–15 min of contact (Figure 10a), as well as the microbial growth and biofilm development on medical device surface, quantified at 24, 48, and 72 h, respectively (Figure 10b).

The initial adhesion was inhibited by the bare Ti and the two BCP coating formulations (Figure 10a), which, thus, demonstrated intrinsic repellent properties. In the case of BCP coatings, the inhibition of bacterial adhesion and colonization could be related to the ability to solubilize in aqueous media, which leads to dynamic surface changes, resulting in major constituents (i.e., Ca and P) and trace elements (e.g., Na, Mg, Si, and/or S) activation.

A similar effect in the case of bare Ti is rather unexpected. The inhibitory effect of Ti could be associated with its hydrophobicity with respect to calcium phosphate PLD layers derived from biogenic resources [66], which generally elicit water contact angles in the hydrophilic domain. However, under conditions of prolonged incubation times, the BCP coatings are capable to limit the biofilm development, to the difference of bare Ti (Figure 10b).

The Spa-BCP coating demonstrated significantly increased anti-biofilm performances after 24, 48, and 72 h of incubation, respectively, as compared to the bare Ti and HA_syn_. The same behavior was also recorded for Sal-BCP, but after 48 and 72 h of incubation only. The one-way ANOVA analysis followed by a Tukey-Kramer post-hoc test indicated that the biofilm development differences recorded for each type of tested sample are statistically significant (*p* < 0.05). The only exception was noticed in the case of HA_syn_ 24 h vs. HA_syn_ 48 h.

It is suggested that the BCP materials, containing a highly soluble component (i.e., β-TCP), may ensure a persistent protection against bacterial colonization for at least 72 h after implantation *via* the release of trace elements (e.g., Na, Mg, Si, and/or S), of which some with already established antimicrobial effects [71,72,73].

## 3. Materials and Methods

### 3.1. Powders Preparation

Spa-BCP and Sal-BCP powders were prepared from *Sparus aurata* (sea bream) and *Salmo salar* (salmon) fish bones. Bones were extracted after gently removing the flesh parts from the entire fish. Remains of fleshy parts on bones were then very carefully cleaned up. The collected bony parts were treated with NaOH (reagent grade) for 24 h, at room temperature, in order to digest the organic moieties. After three repeated ultrasonication washing steps with ethanol and distilled water, followed by drying on a hot plate, the bone pieces were calcined at 850 °C for 4 h in air, to completely eliminate remnant organic traces and alleviate biological hazards, as no microorganisms could withstand such a high temperature. The resulted calcined bone specimens were white-colored. They were next crushed with mortar and pestle into coarse powders, and then grounded for 4 h into fine powders (i.e., with submicron-size particles), using an agate ball mill.

### 3.2. Targets Fabrication

To prepare compact targets, Spa-BCP and Sal-BCP (Marmara University, Nanotechnology and Biomaterials Applications and Research Laboratory, Istanbul, Turkey) and commercial HA_syn_ (Acros Organics B.V.B.A.) powders were pressed at ~6 MPa in a 20 mm diameter mold and sintered for 6 h at 500 °C in air.

### 3.3. Coatings Deposition

PLD experiments were carried out inside a stainless-steel enclosure in water vapors at a residual pressure of 35 Pa. The ablated material was transferred and collected onto chemically etched titanium disks (12 mm diameter, 1.5 mm thick) or (10 × 10) mm^2^ flat silicon wafers. Prior to introduction into the deposition chamber, the substrates were carefully cleaned in an ultrasonic bath Elmasonic X-tra 30H according to the protocol described by Florian et al. [74]. The separation distance between target and substrate was set at 5 cm.

Coatings were synthetized using a pulsed UV KrF* (λ = 248 nm, τ_FWHM_ ≤ 25 ns) COMPexPro 205 F Coherent excimer laser source. To avoid piercing and to obtain a uniform film, the target was continuously rotated during the multi-pulse deposition process with a frequency of 0.75 Hz. The laser beam was incident at 45° onto the target surface and the fluence was set at ≈4.4 J/cm^2^. For the deposition of one film, 15,000 subsequent laser pulses were applied, succeeding to each other with a frequency repetition rate of 10 Hz. During deposition, the substrates were maintained at a constant temperature of 500 °C, by a PID Excel temperature controller. Besides Spa-BCP and Sal-BCP, HA_syn_, and β-TCP_syn_ PLD coatings were also prepared to be used as control samples in the framework of in vitro and/or mechanical assessments.

To improve crystallinity and restore stoichiometry, as-deposited films were submitted to thermal annealing for 6 h at 500 °C, in a water vapors enriched atmospheric air.

### 3.4. Targets and Films Physical–Chemical Analyses

(i) The surface and cross-sectional morphology of PLD films were examined by Scanning Electron Microscopy (SEM) with a FEI Inspect S50 electron microscope (FEI Company, Eindhoven, The Nederlands), operated at 20 kV, under secondary electrons mode. The root mean squared roughness (R_rms_) of films was inferred on the basis of atomic force microscopy (AFM) analysis performed with a TT-Workshop apparatus, in non-contact mode. As the PLD samples were rough, the AFM scanning speed was limited to 0.1 Hz at (450–470) mV and to 0.2 Hz at (470–500) mV, when inspecting film surface of 15 × 15 µm^2^ and 5 × 5 µm^2^, respectively.
(ii) The elemental composition of both PLD targets and films was evaluated by Energy Dispersive X-ray (EDX) spectroscopy, using an EDAX Inc. (Mahwah, NJ, USA) instrument attached to the SEM system and operated at 20 kV. The analysis was performed at least in four randomly chosen SEM (~150 × 150 µm^2^) locations of tested specimens. Collected data were calibrated against National Institute of Standards and Technology (NIST) 2910b HA standard material and presented as mean ± standard deviation (SD).
(iii) The crystalline status of thin films was investigated by X-ray Diffraction (XRD) using a Bruker D8 Advance system (Bruker, Karlsruhe, Germany) with CuK_α_ (λ = 1.5418 Å) radiation. The instrument is equipped with a high efficiency LynxEye^TM^ linear detector and a rotating sample holder (a speed of 30 rot/min was used) to average for compositional non-uniformities. The diffraction patterns were acquired in symmetric (Bragg-Brentano) geometry within the (9–70)° (2θ) angular range, with 0.02° step size and acquisition times of 1 and 8 s per step in the case of PLD targets and deposited films, respectively. The phase composition, average crystallite size, and lattice parameters of the PLD targets and films were inferred *via* structure refinement performed with the MAUD diffraction data processing program (v2.55), by applying the Rietveld whole powder pattern fitting [75]. The “composite crystallite” shape was inferred by applying the Popa approach [53,76] and plotted using the routine embedded in the MAUD software.
(iv) The short-range structural order, bonding architecture and identification of functional groups were studied by Fourier Transform Infra-Red (FTIR) spectroscopy in attenuated total reflectance (ATR) mode. The FTIR-ATR spectra were acquired in the (1600–530) cm^−1^ wave numbers range using a Perkin Elmer Spectrum BX II apparatus (Waltham, MA, USA), equipped with a Pike MIRacle ATR attachment with diamond/zinc selenide crystal. The spectra were average over 32 individual scans performed with a resolution of 4 cm^−1^.
(v) The bonding strength of the CaP-based films was quantified by the pull-off test method, employing a dedicated PAT handy adhesion tester (DFD^®^ Instruments, Kristiansand, Norway), using a testing procedure complying to the ISO 4624:2016 and ASTM D4541—17:2017 standards, and detailed in References [77,78]. Average and SD values were calculated based on triplicate experiments.

### 3.5. In Vitro Biological Assessments of Films

#### 3.5.1. Solubility/Bioactivity Tests under Biomimetic Conditions

The PLD coated samples were immersed in 1 mL of the same cell culture medium (i.e., DMEM supplemented with 10% fetal bovine serum), used in the case of the cytocompatibility assay, detailed here-under. Recent developments have indicated that a more truthful evaluation of the degradation speed/biomineralization capacity of a material can be achieved by testing under cell culture media, and not in the simplistic simulated body fluids such as Tris-HCl or Kokubo SBF, since the cell culture media reproduces with higher accuracy the composition of the intercellular medium [59,64,79]. The solubility/bioactivity in vitro tests were carried out in a dedicated biological incubator under homeostatic conditions (i.e., humidified atmosphere, temperature of 37 °C, and partial pressure of CO_2_ of 5 kPa) for different time periods of 1, 3, and 7 days, respectively. The tests were performed in triplicate. The in vitro evolution of the PLD coatings, in terms of mass loss/gain, was evaluated by weighting with a Radwag MYA 0.8/3.3 Y analytical microbalance, having a readability of 1 μg. Each substrate was weighed prior and after PLD film deposition to estimate the film mass. The weighting was repeated after the sample extraction from the cell culture medium, and subsequent drying for 72 h in an oven at 37 °C. The surface modification of the Spa-BCP and Sal-BCP coatings, which can be induced by dissolution—precipitation processes in the biomimetic medium under homeostatic conditions was explored by SEM at high magnification (100,000×).

#### 3.5.2. Cytocompatibility Tests

##### Cell Culture

Human gingival fibroblasts (HGF-1 cell line, Cat. No. ATCC CRL-2014) were cultured in a complete Dulbecco’s modified Eagle’s medium (DMEM; Invitrogen, Carlsbad, CA, USA) containing 10% fetal bovine serum (Gibco, Life Technologies, Grand Island, NY, USA) at 37 °C in a humidified atmosphere with 5% CO_2_. HGF-1 cells were seeded at a density of 2 × 10^4^ cells/cm^2^ in 24-well plates on the top of PLD coated samples, which were previously sterilized under UV light for one hour. Cells directly cultured on the plastic (polystyrene) surface of the plates were used as control for all in vitro experiments.

##### Microscopic Evaluation of Actin Cytoskeleton

After 24 h culture on the surface of PLD coated samples, the cells were fixed with 4% paraformaldehyde prepared in phosphate-buffered saline (PBS) and the membranes were permeabilized by incubation with a solution of 0.1% Triton X-100 and 2% bovine serum albumin for 1 h at room temperature (RT). The F-actin was then stained for 45 min with 10 µg/mL phalloidin-FITC (fluorescein isothiocyanate) (Sigma-Aldrich, St. Louis, MI, USA) and nuclei were counterstained with 2 µg/mL DAPI (4′,6-diamino-2-phenylindole) for 15 min. The cells were rinsed with PBS and images captured using an inverted fluorescence microscope Olympus IX71 (Tokyo, Japan).

##### Lactate Dehydrogenase (LDH) Cytotoxicity Assay

The culture medium was harvested after 24 h of cell culture on the surface of PLD coated samples, and used to quantify the LDH release with the Cytotoxicity Detection Kit PLUS (Roche Diagnostics Gmbh, Mannheim, Germany), respecting the manufacturer instructions. Volumes of 100 µL culture supernatants were homogenized with 100 µL reaction mixture of catalyst and dye solution, and incubated for 15 min in a dark place at RT. The reaction was stopped by adding 50 µL stop solution, and the absorbance was read at 490 nm using a microplate reader (Flex Station 3, Molecular Devices, San Jose, CA, USA).

#### 3.5.3. Antibacterial Activity Assay

##### Microbial Strains and Growth Conditions

The *Escherichia coli* ATCC 8739 strain was purchased from American Type Culture Collection (ATCC, Manassas, VA, USA). Glycerol stocks were streaked on Tryptone Soy Agar (TSA) (OXOID, Thermo Fisher Scientific) and incubated for 15–18 h to prepare fresh cultures. A microbial inoculum with a density of 1.5 × 10^8^ colony forming units (CFU)/mL, corresponding to the 0.5 McFarland density, was prepared from the fresh culture and then diluted to 1:100.

##### Biofilm Development Assay

The in vitro bacterial adhesion and biofilm development were assessed by using the viable cell counts assay after different times of incubation in the presence of the tested materials. To this purpose, the PLD samples were sterilized by exposure to UV radiation on each side for 30 min, and then placed in a 24-well plate, in 2 mL of sterile saline and inoculated with the microbial suspension, to a final density of ~10^5^ CFU/mL. Samples were incubated at 37 °C for different time periods (T_0_ of 10–15 min, 24, 48, and 72 h, respectively). For each incubation time, a volume of 0.1 mL was extracted from the respective wells and serial ten-fold dilution was performed. A volume of 10 µL of each serial dilution was then plated in duplicate on TSA agar and incubated for 24 h at 37 °C. After incubation, the viable cell counts (VCC) were carried out and the number of CFU/mL for each sample was estimated. All experiments were performed in triplicate.

### 3.6. Statistical Analysis

The statistical analyses were performed using one-way ANOVA followed by a Tukey-Kramer post-hoc test, with the differences being considered significant when *p* < 0.05.

## 4. Conclusions

Pulsed laser deposition technique was extended to the fabrication of bi-phasic calcium phosphates (hydroxyapatite—β-tricalcium phosphate) coatings from sustainable abundant resources of fish bones (sea bream (Spa) and salmon (Sal) species). The transfer of BCPs and the natural doping elements (Na, Mg, Si, and/or S) in form of micron-sized implant-type coatings was probed by both the compositional (EDX) and structural (XRD and FTIR) analyses.

The rise of the β-TCP content was found to augment roughness, decrease bonding strength to Ti substrate, and increase the solubility in vitro in simulated body media of the PLD films.

The synthetized coatings proved efficient against *Escherichia coli* colonization, which is the most frequent Gram-negative species in the etiology of device-associated infections of endogenous origin, while presenting unaltered cytocompatibility with human gingival fibroblast cells with respect to the biological control. Spa-BCP and Sal-BCP coatings demonstrated significantly increased anti-biofilm properties after 24, 48, and 72 h of incubation, respectively, as compared to either the bare Ti substrate or the HA_syn_ coating.

These performances recommend PLD films as a challenging solution for bone regeneration and infections prevention, fit to be integrated into a new generation of biomedical coatings.

## Figures and Tables

**Table 1:** Samples code allocation.

Sample Code	Sample Description
HA_syn_	Commercial HA (Acros Organics B.V.B.A.)
β-TCP_syn_	Commercial beta-tricalcium phosphate (Sigma-Aldrich)
Spa-BCP	Bi-phasic calcium phosphate derived from *Sparus aurata* (sea-bream) fish bones
Sal-BCP	Bi-phasic calcium phosphate derived from *Salmo salar* (salmon) fish bones

**Table 2:** Elemental composition (at. %) of the PLD targets and films, estimated by Energy Dispersive X-ray (EDX) quantitative analyses.

Sample Code	Elemental Composition (at. %)						
	Ca	P	Na	Mg	Si	S	Ca/P Ratio
HA_syn_ target	62.5 ± 0.9	37.5 ± 0.8	–	–	–	–	1.67 ± 0.06
HA_syn_ film	61.4 ± 0.2	38.6 ± 0.3	–	–	–	–	1.59 ± 0.02
Spa-BCP target	58.9 ±3.1	36.2 ± 1.6	2.9 ± 1.1	1.5 ± 0.6	0.2 ± 0.06	0.3 ± 0.07	1.63 ± 0.16
Spa-BCP film	57.7 ± 1.2	38.4 ± 0.7	2.1 ± 0.2	1.1 ± 0.04	0.2 ± 0.03	0.5 ± 0.1	1.50 ± 0.01
Sal-BCP target	60.0 ± 0.7	38.1 ± 0.6	1.0 ± 0.1	0.8 ± 0.1	0.1 ± 0.02	–	1.57 ± 0.04
Sal-BCP film	58.2 ± 0.5	39.5 ± 0.5	1.2 ± 0.2	0.9 ± 0.1	0.2 ± 0.02	–	1.47 ± 0.03

**Table 3:** FTIR-ATR vibration bands assignment.

Wave Number Position (cm^−1^)							IR Band Association
HA_syn_		Spa-BCP		Sal-BCP		β-TCP	
Target	Film	Target	Film	Target	Film		
–	–	562	570	565	572	542	asym. bending (ν_4_) of (PO_4_)^3−^ groups in β-TCP [54,55,56]
561	566					*–*	asym. bending (ν_4_) of (PO_4_)^3−^ groups in HA [5,57,58,59]
–	–	599	600	599	600	600	asym. bending (ν_4_) of (PO_4_)^3−^ groups in β-TCP
599	600					*–*	asym. bending (ν_4_) of (PO_4_)^3−^ groups in HA [5,57,58,59]
629	630	631	629	631	627	*–*	librational mode of (OH)^-^ structural groups in HA [57,58,59]
–	875	–	–	–	–	*–*	out-of-plane (ν_2_) of (CO_3_)^2−^ groups [54,55,56]
–	–	946	942	946	942	944	sym. stretching (ν_1_) of (PO_4_)^3−^ groups in β-TCP [54,55,56]
963	961	963	963	963	963	*–*	sym. stretching (ν_1_) of (PO_4_)^3−^ groups in HA [5,57,58,59]
–	–	983	–	983	–	967	asym. stretching (ν_3_) of (PO_4_)^3−^ groups in β-TCP [54,55,56]
–	–	1022	1024	1015	–	1002	asym. stretching (ν_3_) of (PO_4_)^3−^ groups in β-TCP [54,55,56]
–	–				–	1015	asym. stretching (ν_3_) of (PO_4_)^3−^ groups in β-TCP [5,54,55,56]
1022	1013			1023	1021	*–*	asym. stretching (ν_3_) of (PO_4_)^3−^ groups in HA [5,57,58,59]
1087	1087	1087	1087	1086	1087		asym. stretching (ν_3_) of (PO_4_)^3−^ groups in HA [5,57,58,59]
–	–	1122	–	1123	–	1115	asym. stretching (ν_3_) of (PO_4_)^3−^ groups in β-TCP [54,55,56]
–	1417	–	–	–	–	–	asym. stretching (ν_3_) of (CO_3_)^2−^ groups [57,58,59]
–	1462	–	–	–	–	–	asym. stretching (ν_3_) of (CO_3_)^2−^ groups [57,58,59]
